# Rendering Castor Oil Tasteless

**Published:** 1864-06

**Authors:** 


RENDERING CASTOR OIL TASTELESS.—It is stated by Stan. Martin that the disagreeable taste of this oil may be concealed by beating it well up with the contents of an egg, and adding a little salt or sugar, and a few drops of orange flower water.—*Am. Jour. Pharm.*

